# Social intelligence mediates the protective role of resting-state brain activity in the social cognition network against social anxiety

**DOI:** 10.1093/psyrad/kkae009

**Published:** 2024-04-24

**Authors:** Yingqiao Ma, Yuhan Zou, Xiqin Liu, Taolin Chen, Graham J Kemp, Qiyong Gong, Song Wang

**Affiliations:** Department of Radiology and Huaxi MR Research Center (HMRRC), Functional and Molecular lmaging Key Laboratory of Sichuan Province, West China Hospital, Sichuan University, Chengdu, China; Department of Psychiatry, University of Cambridge, Cambridgeshire, United Kingdom; Department of Radiology and Huaxi MR Research Center (HMRRC), Functional and Molecular lmaging Key Laboratory of Sichuan Province, West China Hospital, Sichuan University, Chengdu, China; Department of Radiology and Huaxi MR Research Center (HMRRC), Functional and Molecular lmaging Key Laboratory of Sichuan Province, West China Hospital, Sichuan University, Chengdu, China; Research Unit of Psychoradiology, Chinese Academy of Medical Sciences, Chengdu, China; Liverpool Magnetic Resonance Imaging Centre (LiMRIC) and Institute of Life Course and Medical Sciences, University of Liverpool, Liverpool L69 3BX, United Kingdom; Department of Radiology, West China Xiamen Hospital of Sichuan University, Xiamen, China; Department of Radiology and Huaxi MR Research Center (HMRRC), Functional and Molecular lmaging Key Laboratory of Sichuan Province, West China Hospital, Sichuan University, Chengdu, China; Research Unit of Psychoradiology, Chinese Academy of Medical Sciences, Chengdu, China

**Keywords:** amplitude of low-frequency fluctuations, social intelligence, social anxiety, resting-state functional magnetic resonance imaging, social cognition network

## Abstract

**Background:**

Social intelligence refers to an important psychosocial skill set encompassing an array of abilities, including effective self-expression, understanding of social contexts, and acting wisely in social interactions. While there is ample evidence of its importance in various mental health outcomes, particularly social anxiety, little is known on the brain correlates underlying social intelligence and how it can mitigate social anxiety.

**Objective:**

This research aims to investigate the functional neural markers of social intelligence and their relations to social anxiety.

**Methods:**

Data of resting-state functional magnetic resonance imaging and behavioral measures were collected from 231 normal students aged 16 to 20 years (48% male). Whole-brain voxel-wise correlation analysis was conducted to detect the functional brain clusters related to social intelligence. Correlation and mediation analyses explored the potential role of social intelligence in the linkage of resting-state brain activities to social anxiety.

**Results:**

Social intelligence was correlated with neural activities (assessed as the fractional amplitude of low-frequency fluctuations, fALFF) among two key brain clusters in the social cognition networks: negatively correlated in left superior frontal gyrus (SFG) and positively correlated in right middle temporal gyrus. Further, the left SFG fALFF was positively correlated with social anxiety; brain–personality–symptom analysis revealed that this relationship was mediated by social intelligence.

**Conclusion:**

These results indicate that resting-state activities in the social cognition networks might influence a person's social anxiety via social intelligence: lower left SFG activity → higher social intelligence → lower social anxiety. These may have implication for developing neurobehavioral interventions to mitigate social anxiety.

## Introduction

Social intelligence refers to a set of psychosocial skills that encompass effective self-expression, understanding social environments, and acting wisely in social interactions (Bar-On, 2006; Barnes and Sternberg, 1989; Petrides, 2011). As a crucial character strength in terms of positive psychology (Peterson and Seligman, 2004), social intelligence is beneficial to personal development and well-being (Avlaev, 2020; Azañedo *et al*., 2020). There is growing evidence that social intelligence is protective against social anxiety, which is a popular mental health problem featured with fear/avoidance of social interaction and performance conditions (American Psychiatric Association, 2013). In particular, social anxiety is related to alterations in the process of self-social information, emotional and social loneliness, and social cognitive patterns (Alvi *et al*., 2022; Harrewijn *et al*., 2017; Wolters *et al*., 2023), all of which overlap with the concept of social intelligence (Petrides, 2011; Silvera *et al*., 2001). There are stable negative associations of social intelligence with social anxiety in several different samples (An and Kochanska, 2021; Chen *et al*., 2023; Hampel *et al*., 2011; Pickard *et al*., 2018).

Despite much research regarding social intelligence at the behavioral level (Walker and Foley, 1973), relatively little is known about its neurobiological underpinnings. As a core aspect of social cognition (Emery *et al*., 2007), social intelligence has been expected to involve brain clusters belonged to social cognition network (SCN), e.g. prefrontal cortex, temporal cortex, amygdala, and insula (Brothers, 1990; Frith, 2007; Kilford *et al*., 2016). An early functional magnetic resonance imaging (fMRI) research identified higher activations in the amygdala, inferior and superior frontal gyrus (IFG/SFG), superior and middle temporal gyrus (STG/MTG), cingulate gyrus, precuneus, and insula during a ‘theory of mind’ task, which tests social intelligence (Baron-Cohen *et al*., 1999). In a structural MRI research of healthy participants, higher social intelligence scores were related to greater gray matter volume (GMV) of bilateral caudate (Myznikov *et al*., 2021). In individuals with autism spectrum disorders, social information processing ability, an important dimension in social intelligence (Silvera *et al*., 2001), has been related to functional connectivity between SFG and the anterior insula (Francis *et al*., 2019). This limited evidence indicates an important role for the SCN in social intelligence. Our first aim in the current research was to examine that relationship using resting-state brain activity in healthy individuals.

The SCN is also implicated in social anxiety. Meta-analyses have demonstrated increased task-based fMRI activity in amygdala, insula, IFG, and STG in SAD patients (Etkin and Wager, 2007), and increased GMV in prefrontal-temporal regions including SFG, IFG, STG, and MTG (Liu *et al*., 2022; Wang *et al*., 2021). There is growing evidence from resting-state studies in SAD of alterations in frontal regions, such as lower activity in SFG and median cingulate gyrus (Brühl *et al*., 2014; Mizzi *et al*., 2022), possibly related to the impaired social cognitive processing. In the healthy population, brain activations in the medial prefrontal cortex, temporal gyrus, and STG during a social norms processing task are positively related to social anxiety (Bas-Hoogendam *et al*., 2020), which reflects the role of these areas in self-referential processing and social cognition, including understanding people's intentions from their actions. A voxel-based morphometry (VBM) research in healthy adolescents revealed a positive link of social anxiety with right MTG GMV, which is an important structure for cognitive processing regarding subjective feeling and emotion (Wang *et al*., 2021). This overlap between regions associated with social anxiety and social intelligence suggests an underlying pathway from the SCN to social intelligence and social anxiety, although the specific mechanism linking brain features to behavior is unclear. Therefore, our second aim of this research was to use a brain–personality–symptom framework (Wang *et al*., 2021) to evaluate whether social intelligence could mediate the linkage of SCN with social anxiety.

To explore the questions, we used resting-state fMRI (RS-fMRI) scanning and well-validated scales on social intelligence and social anxiety. We analyzed resting-state brain activity using the fractional amplitude of low-frequency fluctuation (fALFF) approach (Zou *et al*., 2008); it has good validities and reliabilities (Gao *et al*., 2023; Li *et al*., 2022; Ma *et al*., 2023) and high specificities and sensitivities (Lv *et al*., 2018), and is widely employed to detect brain areas associated with behavioral constructs (Canario *et al*., 2021; Zou *et al*., 2008) and to identify brain activity changes among neuropsychiatric disorders (Ma *et al*., 2023; Qiu *et al*., 2019; Shang *et al*., 2016). Next, we implemented correlation analysis to explore the connections of social intelligence to voxel-wise fALFF across the whole brain, and confirmed this association with prediction analyses. Given the previous literature, we expected to detect this correlation in SCN brain regions (e.g. SFG, IFG, STG, MTG, precuneus, amygdala, and insula). We then tested whether the brain areas associated with social intelligence were linked to social anxiety. Last, we carried out mediation analyses to test the indirect effect of social intelligence on the linkage of fALFF to social anxiety.

We studied students in the adolescent stage, a transition period marked by changes in affection and cognition linked with structural and functional brain reorganization (Konrad *et al*., 2013; Foulkes and Blakemore, 2018). There is a growing evidence of increasing social anxiety among adolescents, increasing their vulnerability to developing SAD (Haller *et al*., 2015; Miers *et al*., 2013, 2014). Thus, our work may throw light on the protective function of social intelligence against social anxiety, and help the development of targeted neurobehavioral interventions to enhance this.

## Methods

### Participants

Our research enrolled 234 normal students, recently graduated from local public high schools, all native Mandarin Chinese speakers who reported no history of neuropsychiatric illness. After three were excluded for incidentally discovered structural brain abnormalities, 231 participants (121 females) were included in the study. Each student was right-handed given the self-reports of Edinburgh Handedness Inventory (Oldfield, 1971) and gave informed written consent before the testing, which was approved by the West China Hospital research ethics committee. This dataset was collected as part of a larger project primarily investigating the neural mechanism underlying personalities, academic success, and mental health in adolescent students (Pan *et al*., 2023b; Wang *et al*., 2018).

### Behavioral measures

#### Tromsø Social Intelligence Scale (TSIS)

This was assessed using the 21-item TSIS (Silvera *et al*., 2001). It has three dimensions (i.e. social awareness, social skills, and social information processing), each comprising seven statements. For each item, participants were asked to indicate how well a statement (e.g. “I find people unpredictable”) describes them, using a seven-point Likert scale from 1 to 7. The total TSIS score (the most useful in empirical research (Savci *et al*., 2022; Swain *et al*., 2022)) sums the ratings of each item, a higher score showing higher social intelligence. This scale has good psychometric properties in adults (Silvera *et al*., 2001) and adolescents (Gini, 2006), and the Chinese version has satisfactory validities and reliabilities (Guo *et al*., 2012; Ling *et al*., 2016; Zhou *et al*., 2014). Cronbach's Alpha for TSIS here was 0.89, evidencing good internal reliability.

#### Liebowitz Social Anxiety Scale (LSAS)

This was evaluated using the 24-item LSAS (Liebowitz, 1987), depicting corresponding situations for each of which participants were asked to indicate, on a scale from 0 to 3, the frequency and degree regarding avoidance and fear. The total score sums the ratings for each item, higher scores representing greater social anxiety. LSAS shows satisfactory psychometric features (Baker *et al*., 2002; Heimberg *et al*., 1999; Oakman *et al*., 2003), and adequate validities and reliabilities among Chinese samples (He and Zhang, 2004; Liao *et al*., 2010; Yang *et al*., 2015). Cronbach's Alpha for LSAS here was 0.93, evidencing satisfied internal reliability.

#### Subjective Socioeconomic Status Scale (SSSS)

As socioeconomic status (SES) plays an important role in brain development (Hackman and Farah, 2009), we adjusted for SES assessed using a single-item scale, which is a diagram of a ladder using 10 rungs (Adler *et al*., 2000). The participants were required to choose a rung to indicate their parents’ situations. Compared with objective measures of SES, the SSSS is more predictive regarding health-linked variables and has been well used among Chinese samples (Lai *et al*., 2020; Liu *et al*., 2023).

### RS-fMRI data collection and analyses

#### Data collection

MRI data were obtained from a Siemens (Erlangen, Germany) Trio 3.0 T MRI scanner, equipped with a 12-channel head coil. We obtained anatomical images (T1-weighted) using these parameters: 176 slices, flip angle 9°, matrix 256 × 256, echo time (TE) 2.26 ms, inversion time (TI) 900 ms, repetition time (TR) 1900 ms, and voxel size 1 × 1 × 1 mm^3^. We then obtained resting images with an echo-planar imaging (EPI) sequence: voxel size 3.75 × 3.75 × 5 mm^3^, 240 volumes;, TE 30 ms, TR 2000 ms, field of view 240 × 240 mm^2^, matrix 64 × 64, interslice gap 0 mm, slice thickness 5 mm, 30 slices, and flip angle 90°. We used foam pads and ear plugs to reduce head motions and noise perception; during resting scans, participants were indicated to lie still, to close their eyes but remain awake, and to not thinking of things on purpose.

#### Data preprocessing

Images were inspected by a clinical radiologist blind to the current study; three students were excluded given neuroanatomical alterations. Image preprocessing, using SPM software and the DPARSF toolbox (Chao-Gan and Yu-Feng, 2010), included: discarding the first 10 images to ensure signal stabilization; correcting slice timing and head motions; realignments; normalizing using 3 × 3 × 3 mm^3^ resolutions; smoothing with an 8 mm full-width at half-maximum Gaussian kernel; removal of linear trends; and computing the mean frame-wise displacement (FD). Then we regressed out six head motioning parameters (Friston *et al*., 1996), as well as the signals of cerebrospinal fluid, white matter, and global mean.

#### fALFF calculation

We computed this measure using the method of Zou *et al*. (2008) via the DPARSF toolbox (Chao-Gan and Yu-Feng, 2010), which is based on the research of Zang *et al*. (2007). The detailed computing processing of this measure can be seen our previous work (Zhang *et al*., 2022).

### Statistical analysis

#### fALFF-behavior correlation analysis

To detect the brain areas where resting activity was linked to social intelligence, we correlated individual social intelligence scores with voxel-wise fALFF in the brain, controlling for gender, age, FD, and family SES scores. Further, we carried out condition-by-covariate interaction analyses (Pan *et al*., 2023b) to test gender difference in the association between social intelligence and fALFF, with age, family SES, and FD as covariates. Gaussian random field theories were used to conduct corrections for the resulting map (Worsley *et al*., 1996; Eickhoff *et al*., 2006), with a voxel-level threshold *P <* 0.01 and cluster-level threshold of *P <* 0.05, as widely applied with resting-state brain imaging research (Cox *et al*., 2012; Wang *et al*., 2022). We conducted these analyses with REST software (Song *et al*., 2011).

#### Confirmatory prediction analysis

As widely used in neuroimaging studies (Lai *et al*., 2020; Qin *et al*., 2014; Supekar *et al*., 2013; Wang *et al*., 2021, 2023; Zhang *et al*., 2022), this was performed to validate the stability of the fALFF-social intelligence connection. Sex, age, FD, and family SES were treated as the controlling variables and the detailed procedure of this analysis can be seen in our previous studies (Lai *et al*., 2020; Wang *et al*., 2021, 2023; Zhang *et al*., 2022).

#### Mapping onto large-scale brain networks

As depicted in our previous research (Liu *et al*., 2023; Pan *et al*., 2023a), we implemented this to map the detected brain regions onto seven key networks: visual network, ventral attention network, somatomotor network, affective network, central executive network, dorsal attention network, and default mode network (DMN) (Yeo *et al*., 2011).

#### Mediation analysis

By using the SPSS macro PROCESS (Hayes, 2017), we performed this to check the indirect effect of social intelligence on the linkage of intrinsic brain activity to social anxiety. In the main analysis, the predict variable (*X*) was resting brain activity, the mediator variable (*M*) was social intelligence, and the outcome variable (*Y*) was social anxiety; the indirect effect is computed as the product of path a (relationship between *X* and *M*) and path *b* (relationship between *M* and *Y* after controlling for X) (Baron and Kenny, 1986). The indirect effect measured the mediation, and to estimate its significance we used bootstrapping procedures (Preacher and Hayes, 2008), in which 5000 bootstrap sampling was used to create 95% confidence interval (CI); if a CI did not contain 0, the indirect effect was significant at *P <* 0.05. Sex, age, FD, and family SES were treated as nuisance variables. To test the directionality of these relations we built an alternative mediation model in which social intelligence was the *X*, social anxiety the *Y*, and resting-state brain activity the *M*.

## Results

### Behavioral results

The descriptive statistics are shown in Table 1. All measures might be normally distributed, with kurtosis and skewness between −1 and + 1 (Marcoulides and Hershberger, 1997). TSIS total scores were highly positively correlated with its three-component dimension scores (social information processing: *r* = 0.84, *P  <* 0.001; social skills: *r* = 0.87, *P  <* 0.001; social awareness: *r* = 0.82, *P <* 0.001), and thus was used as the single measure of social intelligence. Social intelligence did not differ between genders [*t* (229) = 0.01, *P  =* 0.99] or correlate with age (*r* = −0.03, *P =* 0.55), but showed a positive correlation with family SES (*r* = 0.22, *P <* 0.01). There was a negative correlation (*r* = −0.34, *P <* 0.001) between social intelligence and social anxiety.

**Table 1:** Means, SD, ranges, and correlations of age and behavioral constructs.

Variable	Mean	SD	Range	Age	TSIS	LSAS	Family SES
Age	18.5	0.5	16–20	-			
TSIS	98.0	14.0	52–142	−0.03	-		
LSAS	41.1	18.8	6–109	0.05	−0.34**	-	
Family SES	5.1	1.4	1.5–9	−0.06	0.22**	−0.14*	-

*P < 0.05, **P < 0.01.

### Brain regions associated with social intelligence

**Figure 1:**
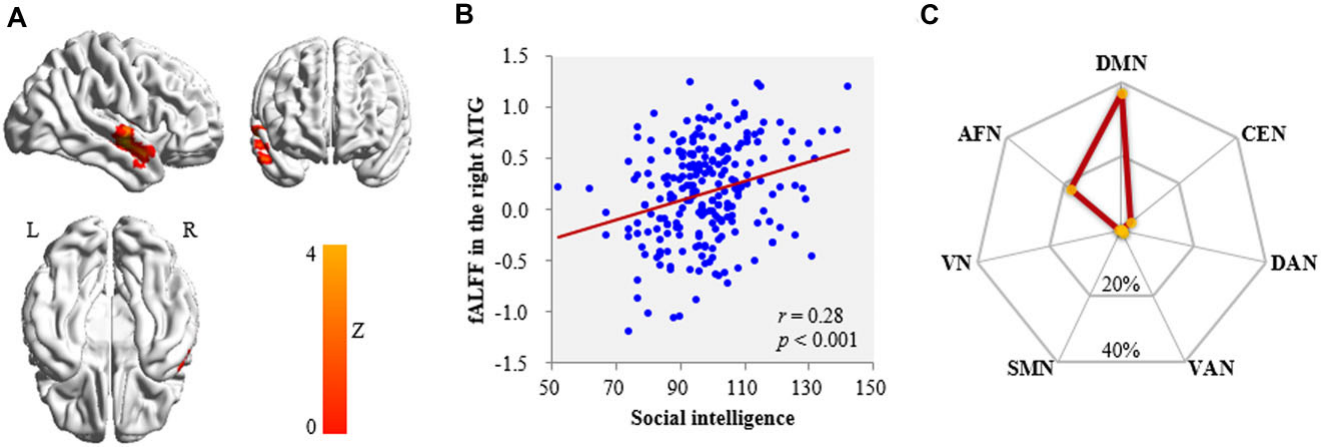
Brain region is positively linked to social intelligence. (**A**) Brain images reveal that social intelligence is positively linked with fALFF in the right MTG after adjusting for gender, age, head motion, and family SES. (**B**) Scatter plots demonstrate the correlation between social intelligence and fALFF in the MTG (*r* = 0.28, *P <* 0.001). (**C**) Plot shows the similarity of co-activation pattern of right MTG to large-scale functional networks. Abbreviations: L, left; R, right; DMN, default mode network; CEN, central executive network; DAN, dorsal attention network; VAN, ventral attention network; SMN, somatomotor network; VN, visual network; AFN, affective network.

**Figure 2:**
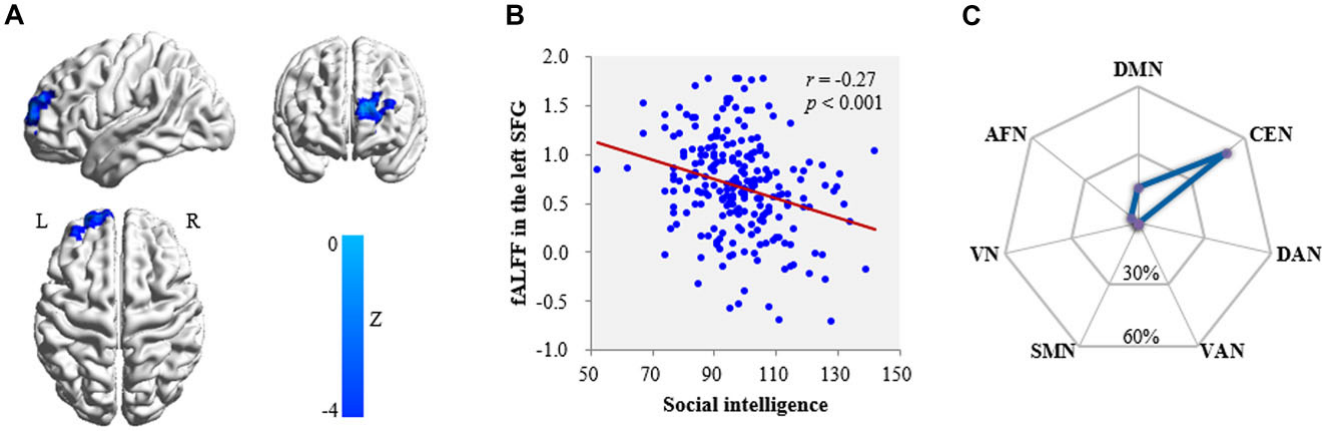
Brain region is negatively linked to social intelligence. (**A**) Brain images reveal that social intelligence is negatively linked with fALFF in the left SFG after adjusting for gender, age, head motion, and family SES. (**B**) Scatter plots demonstrate the correlation between social intelligence and fALFF in the SFG (*r* = −0.27, *P  <* 0.001). (**C**) Plot shows the similarity of co-activation pattern of left SFG to large-scale functional networks. Abbreviations: L, left; R, right; DMN, default mode network; CEN, central executive network; DAN, dorsal attention network; VAN, ventral attention network; SMN, somatomotor network; VN, visual network; AFN, affective network.

Correlation analysis with voxel-wise fALFF (controlling for gender, age, FD, and family SES) found that social intelligence was related to fALFF in two clusters: positively in the right MTG (Fig. 1A and B, Table   2) and negatively in the left SFG (Fig. 2A and B, Table   2). Prediction analyses (with gender, age, FD, and family SES as the covariates) confirmed the stability of these relationships for both right MTG (*r*_[predicted, observed]_ = 0.22, *P <* 0.05) and left SFG (*r*_[predicted, observed]_ = 0.16, *P <* 0.05). In short, higher social intelligence is associated with lower SFG activity and higher left MTG activity. Moreover, condition-by-covariate interaction analyses revealed no significant clusters for the interacting effects of social intelligence with gender.

**Table 2:** Brain regions associated with social intelligence.

		Peak MNI coordinate			Peak *Z* score	Cluster size (voxels)
Region	BA	*X*	*Y*	*Z*		
Right MTG	22	57	−9	−9	3.81	76
Left SFG	10	−15	66	15	−3.89	68

Abbreviations: MNI, Montreal Neurological Institute; BA, Brodmann's Area.

Mapping the MTG onto the large-scale brain networks (Fig. 1C), the most voxels were in the DMN [relative distribution (RD) 37.22%] and affective network (RD 17.54%). Mapping the SFG onto the large-scale intrinsic functional connectivity atlas (Fig. 2C), the most voxels were in the central executive network (RD 49.70%) and DMN (RD 15.62%).

### Relations between social anxiety and brain activity in clusters associated with social intelligence

Having extracted the mean fALFF in these two brain regions we found a positive connection with social anxiety for left SFG (*r* = 0.21, *P <* 0.01), but no correlation for right MTG (*r* = −0.12, *P =* 0.06). Thus, lower levels of social anxiety are related to lower left SFG activities.

### Social intelligence links SFG brain activity and social anxiety

**Figure 3:**
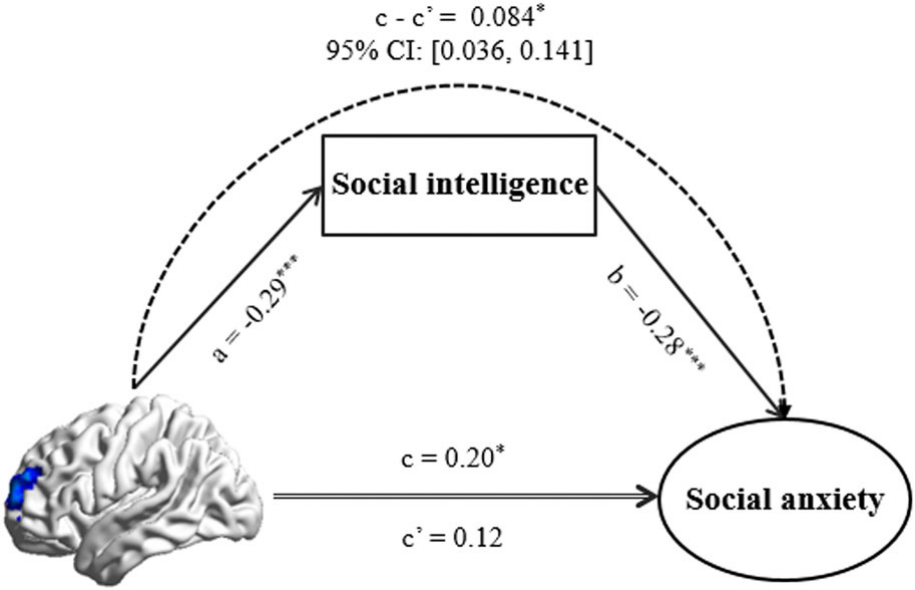
Mediation analysis. Social intelligence mediates the effect of left SFG activity on social anxiety. Standardized regression coefficients are presented in the path diagram. Gender, age, head motion, and family SES are controlled for in the model. ***P < 0.01, *P < 0.05.

Putting together the results of the above, we found that lower SFG activities are linked to both lower social anxiety and higher social intelligence. We were primarily interested in the causal links leading to social anxiety. Applying the brain–personality–symptom analysis described in the Method section, we found that social intelligence (the *M* in the main model) showed significant mediation effects on the connection of the left SFG activity (the *X*) to social anxiety (the *Y*) [the indirect effect = 0.084, 95% CI = (0.036, 0.141), *P <* 0.05], accounting for gender, age, FD, and family SES (Fig. 3). By contrast, in the alternate model, left SFG activity (now the *M*) did not mediate the link of social intelligence (now the *X*) to social anxiety (the common *Y*) [the indirect effect = −0.034, 95% CI = (−0.104, 0.007), *P >* 0.05], accounting for sex, age, FD, and family SES. Thus this evidence supports the model in which resting-state SFG activity in the social cognition network impacts social anxiety via social intelligence, not the alternate model in which social intelligence affects social anxiety via SFG activity.

## Discussion

We set out to examine the brain bases (in terms of regional spontaneous brain activity) of social intelligence, and the potential mediating role of social intelligence in linking spontaneous brain activity to social anxiety. We showed that improved social intelligence was related to lower fALFF in the left SFG and higher fALFF in the right MTG (and vice versa), and that social intelligence mediated the positive linkage between the left SFG fALFF and social anxiety. This study may be the first to define resting-state neurological markers of social intelligence, and throws light on the neurobehavioral mechanism by which social intelligence protects against social anxiety. We discuss these points next.

### Neural correlates of social intelligence

The two regions whose spontaneous activity linked to social intelligence make sense given what is known from other structural and functional brain studies. First, social intelligence was positively associated with fALFF in right MTG. The MTG, bounded dorsally by the STG/superior temporal sulcus and ventrally by the inferior temporal gyrus/inferior temporal sulcus (Jabbour *et al*., 2004), is a core region in the SCN (Diveica *et al*., 2021; Fernández *et al*., 2018; Xu *et al*., 2019; Yun *et al*., 2017), involved in processing social signals related to sound and emotion (Feng *et al*., 2018; Kuhnke *et al*., 2023; Sabatinelli *et al*., 2011). fMRI research has shown MTG activation in theory of mind tasks (Baron-Cohen *et al*., 1999; Diveica *et al*., 2021; Schurz *et al*., 2017), and a positive connection between MTG activation and empathy (Immordino-Yang *et al*., 2009; Kédia *et al*., 2008; Mercadillo *et al*., 2011; Moll *et al*., 2007). Individuals with autistic traits show activation of MTG when processing negative emotion (Yu *et al*., 2020), which are a type of social information (Garrido, 2020). In addition, "mentalizing" (sometimes referred to as "thinking about thinking") a social situation recruits MTG (Veroude *et al*., 2012). A recent meta-analysis reported MTG activation in self-related understanding and perception (e.g. being aware of, obtaining knowledge about, or making judgments toward the self) (Lobo *et al*., 2023). Understandings of self-others' beliefs and emotions and complex social situation information are important dimensions of social intelligence (Kosmitzki and John, 1993).

Second, social intelligence was negatively linked with fALFF of the left SFG, another core region in the SCN (Tuerk *et al*., 2020). The SFG is activated by the tasks of theory of mind (Baron-Cohen *et al*., 1999). Children with autistic spectrum disorder show negative relations between left SFG GMV and social communication ability (Cheng *et al*., 2023). The level of social information processing, a core component of social intelligence (Silvera *et al*., 2001), is predicted by the functional connectivity of SFG and anterior insula (Francis *et al*., 2019). The SFG is involved in social cognition (Chen *et al*., 2018), self-awareness (Goldberg et al., 2006), and emotional regulation (Frank *et al*., 2014). In a social-cognitive task, functional connectivity in the left SFG is increased in the other-situation compared with the self-situation (Ribeiro da Costa *et al*., 2022). Recent meta-analysis has revealed SFG involvement in affiliation and attachment as well as understanding and perception of self and others constructs in social processes (Lobo *et al*., 2023).

### Correlates of social anxiety

The moderate negative behavioral correlation we observed regarding social intelligence and social anxiety is consistent with previous reports (Ashbaugh *et al*., 2005; Hampel *et al*., 2011; Voncken and Bögels, 2008). We also identified a positive relation of social anxiety to fALFF in left SFG. Again, this neural correlate makes sense. There are many reports of SFG functional/structural alterations in SAD patients (Hamilton *et al*., 2015; Liu *et al*., 2022; Qiu *et al*., 2015; Wang *et al*., 2018) and healthy people with increased social anxiety (Smith *et al*., 2019; Kim *et al*., 2023). These include resting state studies: SAD patients show increased intra-network functional network connectivity in the anterior DMN (mainly the SFG) in contrast to healthy controls (Zhang *et al*., 2023); in families genetically enriching for SAD, social anxiety co-segregates with the functional connectivity in the dorsal attention network including SFG (Bas-Hoogendam *et al*., 2021) and altered functional connectivity of SFG and anterior cingulate gyrus, a key circuit of SCN has been suggested as a diagnostically useful biomarker in SAD (Cui *et al*., 2017).

### The mediating role of social intelligence

Thus there is a connection among higher social intelligence, lower left SFG activity, and lower anxiety (and vice versa). If, as we hypothesize, these are causally linked, our mediation analysis gives the direction of causation: social intelligence plays a mediating role between fALFF in the left SFG and social anxiety. This suggests that resting-state activity in the social cognition network might influence a person's social anxiety via social intelligence: lower left SFG activity → higher social intelligence → lower social anxiety.

### Limitations

First, a cross-sectional design cannot draw definitive causal conclusions. Longitudinal studies need to be done. Second, although these behavioral measures are widely used and have satisfied reliabilities and validities (Majid *et al*., 2022; Pepe *et al*., 2021), the self-report aspect may lead to response bias (Lyu and Bolt, 2022). Objective behavioral measures are needed in future research. Third, the participants are healthy high school graduates, a population to vulnerable to social anxiety (Bruce *et al*., 2005), thus our results may not generalize to other samples. Future research should recruit participants with more diversity in age, education, occupation, and mental illness. Last, in our study only SFG and MTG were associated with social intelligence, not other core brain regions in the SCN such as the amygdala, IFG, and STG. This may be due to our use of fALFF, which can only reflect local brain function. Future studies could usefully take a network approach (Hacker *et al*., 2013; Lin *et al*., 2023; Yeo *et al*., 2011).

## Conclusion

This research extends previous investigations by identifying a functional brain marker of social intelligence and revealing a potential “brain-personality-symptom” pathway to protect social anxiety. Specifically, we found that social intelligence was supported by spontaneous activities in the right MTG and left SFG and revealed indirect effects of SFG activity on social anxiety via social intelligence. This research provides an insight into the neurobiological bases linked to social intelligence, and may have significance for underlying neuropsychological markers for the early detection and prevention of social anxiety in adolescents, and for preventive and therapeutic neurobehavioral interventions (Kaminska *et al*., 2020; Paes *et al*., 2013) to reduce the social anxiety of adolescents and improve their mental health.

## Data Availability

To get access to the data and comply with the terms of our research ethics committee approval an application to the corresponding author should be required. West China Hospital has an institutional commitment to data-sharing.
