# Correction to: Social intelligence mediates the protective role of resting-state brain activity in the social cognition network against social anxiety

**DOI:** 10.1093/psyrad/kkae018

**Published:** 2024-09-26

This is a correction to: Yingqiao Ma, Yuhan Zou, Xiqin Liu, Taolin Chen, Graham J Kemp, Qiyong Gong, Song Wang, Social intelligence mediates the protective role of resting-state brain activity in the social cognition network against social anxiety, *Psychoradiology*, Volume 4, 2024, kkae009, https://doi.org/10.1093/psyrad/kkae009

The following change has been made to the originally published manuscript. The following statement has been added to the **Conflict of Interest** section to read: “One of the authors, Qiyong Gong, is also the editor-in-chief of *Psychoradiology*. He was blinded from reviewing or making decisions on the manuscript.” instead of: "The authors declared no potential conflict of interests."

